# Socioeconomic inequalities in mortality from conditions amenable to medical interventions: do they reflect inequalities in access or quality of health care?

**DOI:** 10.1186/1471-2458-12-346

**Published:** 2012-05-11

**Authors:** Iris Plug, Rasmus Hoffmann, Barbara Artnik, Matthias Bopp, Carme Borrell, Giuseppe Costa, Patrick Deboosere, Santi Esnaola, Ramune Kalediene, Mall Leinsalu, Olle Lundberg, Pekka Martikainen, Enrique Regidor, Jitka Rychtarikova, Björn Heine Strand, Bogdan Wojtyniak, Johan P Mackenbach

**Affiliations:** 1 Department of Public Health, Erasmus MC, Rotterdam, Netherlands; 2 Department of Public Health, Faculty of Medicine, Ljubljana, Slovenia; 3 Institute of Social and Preventive Medicine, University of Zurich, Zurich, Switzerland; 4 Agència de Salut Pública de Barcelona, Barcelona, Spain; 5 Regional Epidemiology Unit, ASL TO3 Piedmont Regio, Grugliasco, Italy; 6 Department of Clinical and Biological Sciences, University of Turin, Turin, Italy; 7 Department of Social Research, Vrije Universiteit, Brussel, Brussel, Belgium; 8 Research Unit, Department of Health, Basque Government, Vittoria-Gasteiz, Spain; 9 Lithuanian University of Health Sciences, Kaunas, Lithuania; 10 Stockholm Centre on Health of Societies in Transition, Södertörn University College, Södertorn, Sweden; 11 Department of Epidemiology and Biostatistics, National institute for Health Development, Talinn, Estonia; 12 CHESS, Centre for Health Equity Studies, Stockholm, Sweden; 13 Department of Sociology, University of Helsinki, Helsinki, Finland; 14 Department of Preventive Medicine and Public Health, Faculty of Medicine, Universidad Complutense de Madrid, Madrid, Spain; 15 Department of Demography and Geodemography, Faculty of Science, Charles University, Prague, the Czech Republic; 16 Norwegian Institute of Public Health, Oslo, Norway; 17 National institute of Public Health-National Institute of Hygiene, Warsaw, Poland; 18 Department of Public Health, Erasmus MC, P.O. Box 2030, Rotterdam, CA, 3000, The Netherlands

## Abstract

**Background:**

Previous studies have reported large socioeconomic inequalities in mortality from conditions amenable to medical intervention, but it is unclear whether these can be attributed to inequalities in access or quality of health care, or to confounding influences such as inequalities in background risk of diseases. We therefore studied whether inequalities in mortality from conditions amenable to medical intervention vary between countries in patterns which differ from those observed for other (non-amenable) causes of death. More specifically, we hypothesized that, as compared to non-amenable causes, inequalities in mortality from amenable causes are more strongly associated with inequalities in health care use and less strongly with inequalities in common risk factors for disease such as smoking.

**Methods:**

Cause-specific mortality data for people aged 30–74 years were obtained for 14 countries, and were analysed by calculating age-standardized mortality rates and relative risks comparing a lower with a higher educational group. Survey data on health care use and behavioural risk factors for people aged 30–74 years were obtained for 12 countries, and were analysed by calculating age-and sex-adjusted odds ratios comparing a low with a higher educational group. Patterns of association were explored by calculating correlation coefficients.

**Results:**

In most countries and for most amenable causes of death substantial inequalities in mortality were observed, but inequalities in mortality from amenable causes did not vary between countries in patterns that are different from those seen for inequalities in non-amenable mortality. As compared to non-amenable causes, inequalities in mortality from amenable causes are not more strongly associated with inequalities in health care use. Inequalities in mortality from amenable causes are also not less strongly associated with common risk factors such as smoking.

**Conclusions:**

We did not find evidence that inequalities in mortality from amenable conditions are related to inequalities in access or quality of health care. Further research is needed to find the causes of socio-economic inequalities in mortality from amenable conditions, and caution should be exercised in interpreting these inequalities as indicating health care deficiencies.

## Background

Socioeconomic inequalities in health are one of the main challenges for public health [1]. Systematic inequalities in morbidity and mortality between socioeconomic groups exist in all countries with available data [2], and have not diminished in recent decades. In countries with good time trend data there is even evidence that inequalities in mortality have widened over time [3,4].

One factor that may contribute to health inequalities is lack of access to good quality health care in lower socioeconomic groups. The evidence on this point, however, is inconclusive, particularly for high income countries with publicly financed health care systems. In the latter, lower socioeconomic groups tend to use more care than higher socioeconomic groups in accordance with their higher levels of need [5,6], and although health care outcomes are sometimes less good for patients with a lower socioeconomic status, it is difficult to find direct evidence on the contribution of health care factors to e.g. inequalities in mortality at the population level.

It is for this reason that some studies have looked at inequalities in mortality from conditions considered to be amenable to medical intervention. This approach originates from the Working Group on Preventable and Manageable Diseases led by David Rutstein at Harvard Medical School in the USA in the 1970 s [7] and is based on the idea that deaths from certain causes, and at certain ages, should not occur in the presence of timely and effective health care. Rutstein’s group introduced the notion of ‘unnecessary untimely deaths’ that should be considered as ‘sentinel health events’ and so may provide a marker of the quality of care. This approach has been applied to the study of international and regional variations in mortality [8], and to the study of racial and socioeconomic variations in mortality [9].

For example, we have recently published a study on educational variations in mortality from conditions amenable to medical intervention in 16 European populations. This study showed that inequalities in mortality from these conditions are often substantial, and contribute between 11 and 24% to inequalities in partial life expectancy between the ages of 30 and 64 [10]. This study could, however, not provide certainty about whether these inequalities really reflect inequalities in access or quality of health care services. Given the fact that people from lower socioeconomic groups are exposed to many forms of material and immaterial disadvantage, there is a distinct possibility that inequalities in mortality from conditions amenable to medical intervention are caused by inequalities in the background risk of disease or prognostic factors, instead of being caused by inequalities in health care factors.

We therefore decided to determine whether inequalities in mortality from conditions amenable to medical intervention vary between countries in patterns which differ from those observed for other (non-amenable) causes of death. More specifically, we hypothesized that, as compared to non-amenable causes, inequalities in mortality from amenable causes are more strongly associated with inequalities in health care use, and are less strongly associated with inequalities in common risk factors for disease such as smoking.

## Methods

### Mortality data

Mortality data from 14 European countries were available for this study. These included four Nordic countries (Sweden, Finland, Denmark and Norway), two Western European countries (Belgium and Switzerland), two Southern European countries (Italy and Spain), four Central and Eastern European countries (Poland, Czech Republic, Hungary and Slovenia) and two Baltic countries (Estonia and Lithuania). The data were drawn from national populations, except for Italy (data for Turin city only) and Spain (data for the Madrid and Basque regions, and Barcelona city only). Mortality data for several Central and Eastern European countries and Estonia come from cross-sectional unlinked mortality studies, in which information on socioeconomic position is derived separately from death certificates and census records. Data for the Basque country and Lithuania are derived from a cross-sectional census linked study. Data for other European countries come from longitudinal follow-up studies, in which socioeconomic position as determined during a census has been linked to mortality (for further details see ref [10]). Table 1 gives an overview of the sources of mortality data.

**Table 1:** Descriptive information on databases included in the analyses

**Country**	**Mortality data**			**Survey data**	
	**Type of data**	**Follow-up period**	**No of person yrs at risk**	**Year**	**No. of respondents**
**Western Europe**					
Belgium	Longitudinal	1991–1996	24861015	1997/2001	18481
Switzerland	Longitudinal	1990–2000	27910587	NA	NA
**Nordic countries**					
Sweden	Longitudinal	1991–2000	43042216	2000/2001	11484
Finland	Longitudinal	1990–2000	25874201	1994/’96/’98/’00/’02’04	20371
Denmark	Longitudinal	1996–2000	13926290	2000	16690
Norway	Longitudinal	1990–2000	19956767	2002	6827
**Southern Europe**					
Italy (Turin)	Longitudinal	1991–2001	4873109	1999/2000^†^	118245
Spain (Barcelona)	Longitudinal	1992–2001	8151810		
Spain (Madrid region)	Longitudinal	1996–1997	3663332	2001^†^	20748
Spain (Basque country)	CS* linked	1996–2001	6098457		
**Baltic region**					
Estonia	CS^*^ unlinked	1998–2002	3435255	2002/2004	4376
Lithuania	CS* linked	2000–2002	5156703	1994/’96/’98/’00/’02/’04	11647
**Central and Eastern Europe**					
Slovenia	Longitudinal	1991–2000	9647451	2002	1489
Poland	CS^*^ unlinked	2001–2003	54883245	NA	NA
Czech republic	CS^*^ unlinked	1999–2003	25761450	2002	2476
Hungary	CS^*^ unlinked	1999–2002	21031348	2000/2003	10532

NA Not available.

*CS=Cross-sectional, †Survey data represent the whole of Italy and Spain.

The list of causes of death amenable to medical intervention was based on the original list developed by Rutstein [7] and as also used in Stirbu et al [10], but we added a few additional causes of death based on recent updates [11-14]: colorectal cancer (advances in early detection using colonoscopy and in treatment using chemotherapy), other heart disease (advances in drug treatment) and ischemic heart disease (introduction of beta-blockers and coronary care units). We apply a strict boundary of the health care system by including only actions delivered by those working within the health care sector (including public health agencies, but excluding institutions engaged in intersectoral action). The causes of death selected for the analysis reported here are shown in Additional file 1: Table S 1, with their code numbers according to the Ninth and Tenth revisions of the International Classification of Diseases. As life expectancy in Europe has been rising we applied an age-limit of 75 years (instead of 65 years as in some previous studies).

### Survey data on health care use and behavioural risk factors

Data on health care use and risk factors for disease were obtained from national health or multipurpose surveys (Table 1). Data were available for 12 of the 14 countries for which mortality data were available. Unfortunately, we did not have access to survey data for Switzerland and Poland. All data were nationally representative. Self-reported health care use comprised visits to general practitioners (GP), visits to specialists and/or visits to any doctor in a specified period preceding the interview, and hospital admission in the past year. The use of medication was measured as the use of any types of medicines and the use of prescribed medicine in a specified recall period. The use of preventive health care was captured in questions relating to the attendance of breast and cervical cancer screening, and cholesterol and blood pressure measurements. The specified recall period varied between the countries.

Behavioural risk factors for disease for which data were available in a form that enabled them to be compared across countries were current tobacco smoking and overweight (measured on the basis of self-reported height and weight, with BMI > 25 as a cut-off point). To supplement this information we also studied inequalities in smoking-related causes of death (cancer of buccal cavity, pharynx and oesophagus, cancer of larynx, cancer of trachea, bronchus, lung and COPD) and alcohol-related causes of death (alcoholic cirrhosis of the liver and inflammation of the pancreas, accidental poisoning by alcohol, alcoholic psychosis, dependence, abuse) which we used as proxies for inequalities in exposure to these risk factors (selected ICD codes are given in Additional file 1: Table S 2.

### Socioeconomic classification

In both the mortality and the survey data we used educational level as a measure of socioeconomic position. In order to increase comparability between countries and data sources, educational levels were categorized as ‘lower education’ (no or primary education and lower secondary education) and ‘higher education’ (upper secondary education, and post-secondary/tertiary education).

### Analysis of mortality and survey data

The analysis of data from longitudinal studies with about 10 years of follow-up was limited to people aged 30–74 years at start of follow-up. To create populations with an approximately equal average age at death, the analysis was performed on slightly younger age-groups for studies with a longitudinal design as compared to the cross-sectional studies (35–79 years). For longitudinal studies with a shorter follow-up period the following age limits were used: 35–79 years for Madrid, and 30–79 years for Belgium and the Basque country (Spain).

We estimated the mortality level of each educational group by calculating age-standardized mortality rates (ASMR) using the European population as a standard (WHO). To assess the association between educational level and mortality rates we also calculated relative risks with 95% confidence intervals using Poisson regression adjusting for age and sex. Because previous analyses had shown that international patterns of educational inequalities in mortality are largely similar for men and women [9], and in order to avoid problems with small numbers of deaths, no sex-specific analyses were performed in this study. Inequalities in health care use and behavioural risk factors were estimated using a multivariate logistic regression model. All odds ratios were adjusted for age and sex. The use of health care was also adjusted for self-rated health. The reference category for all relative risks and odds ratios was the highest educational group.

### Association analyses

In order to determine whether inequalities in mortality from conditions amenable to medical intervention are patterned differently from inequalities in mortality from causes of death not amenable by medical interventions, we used scatter plots and calculated Pearson correlations. We also used Pearson correlations to observe whether inequalities in mortality from conditions amenable to medical intervention are associated with inequalities in health care use or behavioural risk factors. A strong correlation was defined as a Pearson correlation below −0.6 or above 0.6. The correlations were only studied if data were available for at least 50% of the countries, therefore we were able to study the following variables: visit to any doctor, use of any medication, hospital admission, blood pressure screening in the last 5 years, cholesterol screening in the last 5 years, overweight, smoking, smoking-related causes of death and alcohol-related causes of death.

### Ethical review

All data used in the analyses have been routinely collected by statistical agencies, and were anonymized before they were supplied to us. Under these conditions, national regulations do not require ethical review of study proposals.

## Results

### Inequalities in mortality

Table 1 shows the specifics of the mortality databases and the survey data used in this report. For 9 countries longitudinal mortality data (in the case of Italy and Spain limited to specific regions), and for 5 others cross-sectional mortality data were available. For 12 countries survey data were available on use of health care and behavioural risk factors.

Table 2 gives an overview of educational inequalities in mortality from all causes of death and all non-amenable and amenable causes of death. Inequalities in total mortality are the largest in the Baltic region and in Central and Eastern Europe, and the same applies to inequalities in mortality from non-amenable and amenable causes. For example, in Poland the lower educated have a 1.87 (95% CI: 1.86–1.88) fold increased risk of all cause mortality, a 1.85 (95% CI 1.84–1.87) fold increased risk of mortality from non-amenable causes, and a 1.88 (95% CI: 1.86–1.89) fold increased risk of mortality from amenable causes. On the other hand, in Southern European populations the inequalities in mortality tend to be the smallest. Inequalities in amenable mortality are often somewhat larger than inequalities in non-amenable mortality, particularly in Belgium, Sweden, Finland, Norway, the Czech Republic and Hungary, but the reverse also applies, particularly in Southern Europe.

**Table 2:** Numbers of deaths, age standardized mortality rates, and relative risks comparing lower to higher educational level for total, amenable and non-amenable mortality, by country

**Country**		**Total mortality**			**Non amenable causes**		**Amenable causes of death**	
	**No. of deaths**	ASMR*		**RR (CI95)**^**‡**^	**Nr of deaths**	**RR (CI95)**^**‡**^	**Nr of deaths**	**RR (CI95)**^**‡**^
		**Lower**^**†**^**education**	**Higher**^**†**^**education**					
**Western Europe**								
Belgium	283349	1101.04	879.33	1.35 (1.33–1.36)	219916	1.35 (1.33–1.37)	63433	1.35 (1.33–1.37)
Switzerland	255275	928.42	822.37	1.35 (1.34–1.37)	151351	1.34 (1.33–1.36)	103924	1.37 (1.35–1.39)
**Nordic countries**								
Sweden	393038	910.59	711.21	1.30 (1.29–1.31)	211683	1.23 (1.22–1.24)	181355	1.39 (1.38–1.40)
Finland	270232	1208.20	900.75	1.38 (1.36–1.39)	138377	1.33 (1.32–1.35)	131855	1.45 (1.43–1.47)
Denmark	136064	1229.15	945.3	1.32 (1.31–1.34)	88658	1.32 (1.30–1.33)	47406	1.34 (1.31–1.37)
Norway	213022	1131.82	870.68	1.35 (1.34–1.36)	109988	1.30 (1.29–1.32)	103034	1.40 (1.38–1.42)
**Southern Europe**								
Italy (Turin)	50621	966.18	844.96	1.27 (1.24–1.30)	32995	1.29 (1.25–1.33)	17626	1.24 (1.19–1.29)
Spain (Barcelona)	77101	1012.27	949.97	1.23 (1.21–1.25	49540	1.26 (1.24–1.29)	27561	1.19 (1.15–1.22)
Spain (Madrid region)	22588	730.66	706.06	1.22 (1.17–1.26)	14546	1.25 (1.19–1.30)	8042	1.17 (1.11–1.25)
Spain (Basque country)	41813	631.02	666.00	1.09 (1.06–1.12)	27215	1.12 (1.08–1.16)	14598	1.04 (1.00–1.09)
**Baltic region**								
Estonia	60873	1914.96	1169.01	1.43 (1.40–1.45)	27955	1.40 (1.36–1.43)	32918	1.45 (1.42–1.49)
Lithuania	78399	1883.58	939.51	1.67 (1.64–1.88)	37613	1.70 (1.66–1.74)	40786	1.66 (1.62–1.70)
**Central and Eastern Europe**								
Slovenia	101557	1242.52	1082.34	1.43 (1.41–1.45)	57021	1.45(1.43–1.48)	44536	1.41 (1.38–1.44)
Poland	717743	1288.73	704.56	1.87 (1.86–1.88)	397948	1.85 (1.84–1.87)	319795	1.88 (1.86–1.89)
Czech republic	344973	1188.16	690.26	1.92 (1.91–1.94)	178032	1.85 (1.83–1.88)	166941	2.01 (1.98–2.03)
Hungary	368029	1548.94	869.52	1.93 (1.91–1.94)	191651	1.86 (1.83–1.88)	176378	2.00 (1.98–2.03)

*ASMR=age standardized mortality rate per 100.000 person-years.

† Lower education is a combination of primary or no education and lower secondary education, higher education is a combination of upper secondary education and tertiary education.

‡ RR=relative risk adjusted for age and sex.

**Figure 1:**
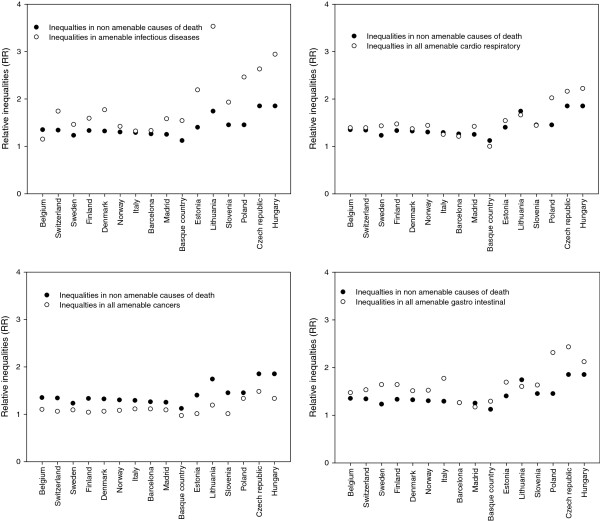
International pattern of inequalities in amenable and non-amenable mortality.

Figure 1 illustrates the patterns of variation for inequalities in amenable and non-amenable mortality, for four groups of amenable causes: all infectious diseases, all amenable cancers, all amenable cardiorespiratory conditions, and all amenable gastrointestinal conditions. While inequalities in mortality from infectious diseases, cardiorespiratory conditions and gastrointestinal conditions tend to be larger than inequalities in non-amenable mortality, inequalities in mortality from cancer tend to be smaller. Both for non-amenable and amenable causes, inequalities in mortality tend to be larger in the Baltic region and in Central and Eastern Europe. Further details on inequalities in amenable mortality can be found in Additional file 1: Table S 3.

As seen in Table 3, positive associations between non-amenable and amenable mortality are found for most amenable causes. Pearson correlations are often above 0.6. The main exceptions are cervical cancer (for which a negative association is found) and other heart disease and cholecystitis and -lithiasis (for which the Pearson correlations are very low).

**Table 3:** Correlation between inequalities in amenable mortality and inequalities in mortality from all causes and from non-amenable causes

**Cause of death**	**Correlation between inequalities in amenable mortality and inequalities in all-cause mortality (Pearson r)**	**Correlation between inequalities in amenable mortality and inequalities in mortality from non-amenable causes (Pearson r)**
**Infectious diseases**		
All infectious diseases	0.832*	0.858*
Tuberculosis (TB)	0.812*	0.708*
Pneumonia/influenza	0.921*	0.921*
Other infectious disease	0.432	0.509*
**Cancer**		
All amenable cancers	0.595*	0.789*
Cervical cancer	−0.410	−0.344
Testicular cancer	0.587*	0.726*
Colorectal cancer	0.472	0.601*
Hodgkin’s disease and leukaemia	0.357	0.553*
**Cardiorespiratory conditions**		
All amenable cardiorespiratory conditions	0.969*	0.872*
Ischaemic heart disease	0.907*	0.822*
Cerebrovascular disease	0.952*	0.889*
Chronic rheumatic heart disease	0.611*	0.554*
Hypertension	0.798*	0.608*
Asthma	0.731*	0.588*
Other heart disease	0.125	0.161
**Gastro intestinal conditions**		
All amenable gastrointestinal conditions	0.853*	0.729*
Appendicitis, hernia, peptic ulcer	0.873*	0.766*
Cholecystitis and -lithiasis	0.316	0.166

*Significant correlation, p < 0.05.

Analysis based on data in Table 2.

### Inequalities in determinants of mortality

Table 4 shows that in many countries the lower educated are less likely to visit a specialist, or to visit any doctor, after taking into account their generally worse health status. Inequalities in visits to any doctor tend to be larger in the Baltic region and in Central and Eastern Europe. Educational inequalities in visits to general practitioners and in hospital admissions are usually small. In some countries the use of medication was lower in lower educated groups, and inequalities in the use of preventive health care (cholesterol, blood pressure, and cervical cancer screening) are found in almost all countries for which these data were available.

**Table 4:** Educational inequalities in use of health care comparing low educational to high educational level

**Country**	Visit GP*	Visit specialist*	Visit any doctor*	Hospital admission*	Use of any medication*	Use of prescribed medicine*	**Cholesterol screening in last 5 years**	**Blood pressure screening in the last 5 years**	**Cervical cancer screening**
	OR (CI95)**	OR (CI95)^**^	OR (CI95)^**^	OR (CI95)^**^	OR (CI95)^**^	OR (CI95)^**^	OR (CI95)^**^	OR (CI95)^**^	OR (CI95)^**^
**Western Europe**									
Belgium	1.14 (1.09–1.20)	0.73 (0.69–0.77)	0.95 (0.91–1.00)	0.99 (0.90–1.11)	0.82 (0.78–0.86)	1.15 (1.07–1.24)	0.69 (0.66–0.72)	0.73 (0.66–0.79)	0.55 (0.50–0.61)
Switzerland	NA	NA	NA	NA	NA	NA	NA	NA	NA
**Nordic countries**									
Sweden	NA	NA	0.83 (0.73–0.94)	NA	NA	NA	NA	NA	NA
Finland	NA	NA	0.78 (0.73–0.84)	NA	NA	NA	0.60 (0.53–0.68)	0.71 (0.56–0.91)	NA
Denmark	0.88 (0.80–0.97)	0.64 (0.54–0.76)	0.84 (0.76–0.92)	1.06 (0.88–1.28)	0.92 (0.84–1.02)	1.08 (0.93–1.24)	0.85 (0.77–0.94)	0.88 (0.80–0.98)	NA
Norway	1.00 (0.82–1.24)	0.70 (0.56–0.87)	0.96 (0.78–1.19)	1.13 (0.89–1.44)	1.20 (0.97–1.47)	1.07 (0.86–1.32)	NA	NA	NA
**Southern Europe**									
Italy	1.03 (0.98–1.08)	0.72 (0.69–0.76)	0.87 (0.83–0.90)	1.05 (0.98–1.13)	0.83 (0.81–0.87)	1.21 (1.13–1.31)	0.84 (0.82–0.87)	0.82 (0.79–0.85)	0.70 (0.68–0.73)
Spain	NA	NA	0.91 (0.82–1.01)	0.88 (0.75–1.02)	0.90 (0.83–0.98)	NA	NA	NA	NA
**Baltic region**									
Estonia	0.87 (0.74–1.02)	0.74 (0.63–0.86)	0.73 (0.62–0.87)	1.10 (0.92–1.33)	1.09 (0.92–1.27)	NA	0.83 (0.70–0.98)	0.72 (0.50–1.02)	0.55 (0.44–0.69)
Lithuania	NA	NA	0.74 (0.67–0.82)	NA	NA	NA	0.97 (0.88–1.07)	0.69 (0.61–0.78)	NA
**Central and Eastern Europe**									
Slovenia	NA	NA	0.79 (0.56–1.12)	NA	NA	NA	NA	NA	NA
Poland	NA	NA	NA	NA	NA	NA	NA	NA	NA
Czech Republic	NA	NA	NA	0.97 (0.78–1.12)	0.66 (0.54–0.81)	1.58 (1.23–2.03)	0.79 (0.70–0.90)	1.07 (0.94–1.22)	0.62 (0.48–0.81)
Hungary	0.80 (0.71–0.89)	0.55 (0.49–0.61)	0.65 (0.58–0.75)	1.00 (0.89–1.13)	0.65 (0.59–0.73)		0.59 (0.55–0.65)	0.67 (0.55–0.86)	NA

*Adjusted for age, sex and self rated health, **(OR CI95)=odd ratio with 95% confidence interval’ In the calculation of the odds ratio the highest educational level is the reference group.

For Switzerland and Poland no survey data were available.

As seen in Table 5, people from lower educational groups are more likely to smoke and to be overweight, although inequalities for the latter risk factor tend to be smaller and less consistent than for the first. Inequalities in mortality from smoking-related causes confirm that inequalities in smoking are larger in Central and Eastern and smaller in Southern Europe. Inequalities in mortality from alcohol-related causes also tend to be larger in Central and Eastern European countries.

**Table 5:** Inequalities in smoking and overweight in European countries between low and high educational groups

**Country**	Smoking^*^	**Overweight**	**Smoking-related deaths**^**‡**^	**Alcohol-related deaths**^**∥**^
	**OR (CI95)**^**†**^	**OR (CI95)**^**†**^	**RR (CI95)**^**†**^	**RR (CI95)**^**†**^
**Western Europe**				
Belgium	1.51 (1.45–1.58)	1.51 (1.45–1.57)	1.72 (1.68–1.77)	1.44 (1.33–1.56)
Switzerland	NA	NA	NA	NA
**Nordic countries**				
Sweden	1.26 (1.13–1.40)	0.96 (0.87–1.05)	1.33 (1.31–1.35)	1.78 (1.72–1.84)
Finland	1.29 (1.12–1.36)	1.07 (1.02–1.13)	1.78 (1.73–1.84)	1.67 (1.61–1.74)
Denmark	1.45 (1.35–1.56)	1.25 (1.16–1.34)	1.42 (1.38–1.46)	1.32 (1.25–1.39)
Norway	1.43 (1.25–1.64)	0.95 (0.83–1.09)	1.57 (1.53–1.63)	1.76 (1.65–1.89)
**Southern Europe**				
Italy	1.08 (1.05–1.11)	1.51 (1.45–1.57)	1.21 (1.16–1.27)	2.31 (1.63–3.47)
Spain	1.21 (1.13–1.29)	NA	1.43 (1.37–1.49)	1.87 (1.42–2.47)
**Baltic region**				
Estonia	1.27 (1.11–1.45)	0.95 (0.83–1.08)	1.64 (1.54–1.74)	1.88 (1.70–2.07)
Lithuania	1.48 (1.35–1.62)	1.03 (0.95–1.08)	2.21 (2.09–2.34)	2.78 (2.53–3.07)
**Central and Eastern Europe**				
Slovenia	1.40 (1.09–1.81)	1.11 (0.86–1.42)	1.52 (1.47–1.58)	2.41 (2.26–2.57)
Poland	NA	NA	NA	NA
Czech Republic	1.86 (1.62–2.12)	1.27 (1.12–1.14)	2.41 (2.34–2.47)	2.69 (2.52–2.87)
Hungary	1.88 (1.72–2.05)	1.21 (1.12–1.32)	2.09 (2.04–2.14)	2.27 (2.19–2.33)

*Smoking= current regular smoking and occasional smoking, Overweight=body mass index > 25.

† OR (CI95)=odds ratio with 95% confidence interval adjusted for age and sex.

‡Smoking related death are chronic obstructive pulmonary disease and cancer of the buccal cavity, pharynx, oesophagus, larynx, trachea, bronchus, and lung.

∥Alcohol related deaths are accidental poisoning by alcohol and alcoholic psychosis, dependence, abuse, cardiomyopathy, cirrhosis of the liver and inflammation of the pancreas.

For Switzerland and Poland no survey data were available (NA).

### Association between inequalities in mortality and inequalities in determinants

Table 6 shows the Pearson correlations between inequalities in amenable mortality and inequalities in both use of health care and behavioural risk factors (details on correlations for specific causes of amenable mortality can be found in the (Additional file 1: Table S 4). A negative correlation was found between inequalities in visit to any doctor and inequalities in mortality from many amenable causes of death, particularly for infectious diseases and all amenable cardio respiratory conditions. In this case a negative correlation implies that larger inequalities in health care use (i.e. greater underutilization in lower educational groups, as indicated by an odds ratio farther below 1) go together with larger inequalities in amenable mortality. However, an equally strong negative correlation was also observed between inequalities in visit to any doctor and inequalities in mortality from non-amenable causes. Inequalities in hospital admission were not associated with inequalities in amenable mortality. Inequalities in use of any medicine were only significantly correlated to inequalities in mortality from amenable cancers.

**Table 6:** **Correlation between inequalities in amenable mortality, inequalities in use of health care**^**†**^**and inequalities in behavioural risk factors**

**Cause of death**	**Use of health care**					**Behavioural risk factors**			
	**Visit any doctor**	**Hospitalization**	**Use of medication**	**Cholesterol screening**	BP^**^screening	**Smoking**	**Overweight**	**Smoking related deaths**	**Alcohol related deaths**
Number of observations	11	8	8	8	8	12	11	14	14
Non amenable causes of death	−0.745*	−0.213	−0.704	−0.059	0.175	0.859*	0.059	0.806*	0.632*
All amenable infectious diseases	−0.762*	−0.010	−0.530	0.297	−0.095	0.637*	−0.209	0.603*	0.659*
All amenable cancers	−0.334	−0.443	−0.752*	−0.071	0.604	0.705*	0.203	0.578*	0.613*
All amenable cardiorespiratory conditions	−0.731*	−0.096	−0.613	−0.289	0.214	0.916**	−0.022	0.851*	0.463
All amenable gastrointestinal conditions	0.506	−0.107	−0.699	−0.302	0.642	0.684*	0.326	0.752*	0.443

† Health care factors are included in the correlation analyses if these were available for at least half of the countries.

*Significant correlation, p < 0.05.

**Blood pressure screening.

As seen in Table 6, consistently positive associations are found between inequalities in amenable mortality and inequalities in smoking or smoking-related mortality. In this case a positive correlation implies that larger inequalities in smoking (as indicated by an odds ratio farther above 1) go together with larger inequalities in amenable mortality. As expected, a positive correlation is also observed between inequalities in smoking and smoking-related mortality and inequalities in mortality from non-amenable causes. As compared with non-amenable causes, inequalities in amenable causes are not less strongly associated with inequalities in smoking or smoking-related deaths. Positive associations are also seen between inequalities in amenable mortality and inequalities in alcohol-related deaths, but not with inequalities in overweight.

## Discussion

### Summary of findings

In most countries and for most amenable causes of death substantial inequalities in mortality were observed, but inequalities in mortality from amenable causes did not vary between countries in patterns that could be distinguished from those seen for inequalities in non-amenable mortality. More specifically, our hypothesis that, as compared to non-amenable causes, inequalities in mortality from amenable causes are more strongly associated with inequalities in health care use and less strongly with inequalities in common risk factors for disease such as smoking, was not supported by the data. Inequalities in mortality from amenable causes are larger in countries with larger inequalities in visits to any doctor, but so are inequalities from non-amenable causes. And just like inequalities in mortality from non-amenable causes, inequalities in mortality from amenable conditions also tended to be larger in countries where inequalities in smoking, smoking-related deaths and alcohol-related deaths were larger.

### Limitations

This study has several limitations. As shown by the variation between studies in selection of causes of death deemed to be amenable to health care intervention [15] this selection is to some extent arbitrary. Our selection is somewhat wider than that used in previous studies, including the study by Stirbu [10] to which this is a follow-up. Because our results are rather consistent across amenable causes of death, we do not think that other selections would have led to substantially different results.

Specific attention should be paid to mortality from HIV/AIDS, which was largely missing from our data. It is only after about 1995 that HIV/AIDS was distinguished as a cause of death. Due to this exclusion, inequalities in mortality from infectious diseases may have been underestimated. This underestimation may be particularly large for southern European countries, which were severely affected by the AIDS/HIV epidemic in the 1990s. It is therefore of special interest to have the results for the Basque country, the only Southern population for which HIV/AIDS deaths were included. Compared to Barcelona, Madrid and Turin, larger relative inequalities in mortality from other infectious diseases were found in the Basque country (Additional file 1: Table S 3).

Our study had to rely on routinely collected mortality data, which are not necessarily fully comparable between countries. Differences between countries in certification and coding of causes of death are unlikely to have affected our results, because our focus is on comparing lower and higher educated groups within the same country. Our results would only be biased if there is an association between country-specific certification or coding practices and educational level, which is not likely. Most of the mortality data used in this study were census-linked, but data from some Central and Eastern European countries and the Baltic region were based on a cross-sectional unlinked design. This limitation has been discussed in the paper of Stirbu et al [10] and is not likely to substantially affect our results.

For most of the determinants our study had to rely on national health or multipurpose surveys, and self-reported data on health care use and behavioural risk factors may be unreliable. The different national surveys varied in phrasing of the questions, answer categories, recall periods and response rates. However, the influence of these differences on the outcome of this study can be expected to be limited. Inequalities in reporting between socioeconomic groups will only have affected our results if these inequalities also differed between countries, which is less likely. Also, a comparison between inequalities in self-reported smoking and mortality from smoking-related causes (Table 5) shows a reasonable degree of correspondence. The limited number of countries available for our correlation analysis also limits the scope of our analysis.

Unfortunately, the number of determinants of mortality that could be included in our analysis was limited. More extensive or more detailed data on inequalities in health care use might have led to different findings, because previous studies have shown that there are socioeconomic inequalities in use of specific medical procedures in many countries [16-19]. However, comparable information on inequalities in application of specific interventions is not available.

### Interpretation

In accordance with previous studies [9,20] we found that a lower educational level is associated with a substantially higher mortality from causes thought to be amenable to medical intervention. These inequalities are particularly large in Central and Eastern Europe and the Baltic region, and have led to speculations about the role of inequalities in access or quality of health care in explaining the large inequalities in all-cause mortality in these countries [21-23]. The current study, however, has not found clear evidence for a role of health care, and suggests that larger inequalities in amenable mortality may instead be due to the same risk factors as those involved in other causes of death.

Many authors have commented on the possibility that observed variations in amenable mortality (between countries, regions, social groups) may be due to variations in background risk, and not in access or quality of health care. It is easy to see that this might be the case, because levels of cause-specific mortality are not only influenced by survival rates but also by the incidence rates of the underlying diseases. Incidence rates of conditions amenable to medical intervention may differ between socioeconomic groups, because they differ in exposure to the determinants of incidence (e.g. material living conditions, behavioural risk factors, psychosocial conditions). Our finding that lower educational groups have a higher prevalence of negative health behaviour, such as smoking, obesity and excessive alcohol use, is in line with what others have found [24-26]. It is interesting to see that in our study the largest inequalities were found in mortality from infectious diseases, e.g. tuberculosis. Although we cannot exclude the possibility that survival from TB is lower among the lower educated, due to inequalities in access or quality of health care, it is well-known that incidence is also higher [27]. In the Baltic countries the high inequalities in mortality from TB might also be explained by alcohol use, which has been shown in an earlier study [28].

We did find inequalities in the use of health care. In a series of studies, Van Doorslaer and colleagues found that inequalities in seeing a specialist are usually “pro-rich”, while seeing a general practitioner is often not related to socio-economic position (after taking health status into account) [29]. Our results (Table 4) are in line with their findings. Previous studies have also reported on a lower use of preventive interventions in lower socioeconomic groups [18,19], a finding that our study reproduced. A lower use of treatment and control of cardiovascular risk factors has also been found for uninsured adults, who tend to come from the lower socioeconomic groups [30].

Although we did find an association between inequalities in health care use (i.e., visit to any doctor) and inequalities in amenable mortality, we regard this as potentially spurious because the same association emerged for inequalities in mortality from non-amenable conditions (Table 6). Our study is one among many which has been unable to find consistent and/or exclusive associations between amenable mortality and health care use [31,32]. Lack of clear evidence of an effect should not be misinterpreted as evidence for lack of an effect, but as the burden of proof lies with those who promote the use of amenable mortality our study urges for caution in the use of these indicators.

Although our findings do not point into the direction of health care, in-depth studies using a more powerful research design may reveal that variations in exposure to shortcomings in health care explain some of the differences in mortality between educational groups. This could be done through a retrospective audit into one specific cause of amenable mortality e.g. TB, colorectal cancer, or cerebrovascular disease. A more ambitious approach would be the performance of a prospective follow up study of one of the amenable conditions, relating socioeconomic variations in mortality outcomes to socioeconomic variations in health care utilization.

## Conclusions

We did not find clear evidence that inequalities in mortality from amenable conditions are related to inequalities in access or quality of health care. Further research is needed to find the causes of socioeconomic inequalities in mortality from amenable conditions, and caution should be exercised in interpreting these inequalities as indicating health care deficiencies.

## Competing interests

The authors declare to have no competing interests.

## Author contributions

IP carried out the analyses, and write a first draft of the paper. RH advised on the analysis. BA, MB, CB, GC, PD, SE, RK, ML, OL, PM, ER, JR, BHS, and BW collected the data. JM conceived the study, and edited the manuscript. All authors commented on the manuscript, and approved the final version.

## Pre-publication history

The pre-publication history for this paper can be accessed here:

http://www.biomedcentral.com/1471-2458/12/346/prepub

## Supplementary Material

Additional file 1 **Table S1.** Causes of death diseases amenable to medical intervention according to the 9th and 10th revisions of the International Classification of Diseases, Clinical Modification (ICD-9-CM and ICD-10 CM). **Table S2.** Smoking- and alcohol-related causes of death according to the 9th and 10th revisions of the International Classification of Diseases, Clinical Modification (ICD-9-CM and ICD-10 CM). **Table S3.** Relative risks comparing low educational level to high educational level for causes of amenable mortality by country. **Table S4.** Correlation between inequalities in causes of amenable mortality and inequalities in use of health care. Click here for file
