# Supplementary material for: Socioeconomic inequalities in mortality from conditions amenable to medical interventions: do they reflect inequalities in access or quality of health care?
Source: BMC Public Health. 2012 May 11;12:346. doi: 10.1186/1471-2458-12-346 (PMC3413570; doi:10.1186/1471-2458-12-346)
Supplement: Additional file 1 — Table S1. Causes of death diseases amenable to medical intervention according to the 9th and 10th revisions of the International Classification of Diseases, Clinical Modification (ICD-9-CM and ICD-10 CM). Table S2. Smoking- and alcohol-related causes of death according to the 9th and 10th revisions of the International Classification of Diseases, Clinical Modification (ICD-9-CM and ICD-10 CM). Table S3. Relative risks comparing low educational level to high educational level for causes of amenable mortality by country. Table S4. Correlation between inequalities in causes of amenable mortality and inequalities in use of health care. [file 1471-2458-12-346-S1.doc]

**Table S1:** Causes of death diseases amenable to medical intervention according to the 9^th^ and 10^th^ revisions of the International Classification of Diseases, Clinical Modification (ICD-9-CM and ICD-10 CM)

Table S2: Smoking- and alcohol-related causes of death according to the 9^th^ and 10^th^ revisions of the International Classification of Diseases, Clinical Modification (ICD-9-CM and ICD-10 CM)

**Table S3**: Relative risks comparing low educational level to high educational level for causes of amenable mortality by country

**Table S4**: Correlation between inequalities in causes of amenable mortality and inequalities in use of health care

**Table S1.** Causes of death diseases amenable to medical intervention and their code numbers according to the 9^th^ and 10^th^ revisions of the International Classification of Diseases, Clinical Modification (ICD-9-CM and ICD-10 CM)

| **Cause of death** | **ICD-9** | **ICD-10** |
| --- | --- | --- |
| **Infectious diseases** |  |  |
| Tuberculosis (TB) | 010-018, 137 | A15-A19 |
| Pneumonia/Influenza | 487;480-486 | J10-J18 |
| Other infectious diseases | Rest(001–139) | Rest(A00-B99) |
| **Cancer** |  |  |
| Cervical cancer | 180 | C53 |
| Testicular cancer | 186 | C62 |
| Colorectal cancer | 153-154 | C18-C21 |
| Hodgkin’s disease and leukaemia | 201,204-208 | C81, C91-95 |
| **Cardiorespiratory conditions** |  |  |
| Ischaemic heart disease | 410-414 | I20-I25 |
| Cerebrovascular disease | 430-438 | I10-15, I60-69 |
| Chronic rhe**u**matic heart disease | 390-398 | I00-09 |
| Hypertension | 401-405 |  |
| Asthma | 493 | J45-46 |
| Other heart disease | 416,420-429 | I26-I52, I98 |
| **Gastrointestinal conditions** |  |  |
| Appendicitis, hernia, peptic ulcer | 531-534, 540–543, 550–553,560 | K25-28, K35-38, K40-46, K56 |
| Cholecystitis and -lithiasis | 574-576 | K80-83 |

**Table S2**: Smoking- and alcohol-related causes of death and their code numbers according to the 9^th^ and 10^th^ revisions of the International

**Classification of Diseases, Clinical Modification (ICD-9-CM and ICD-10 CM)**

| **Cause of death** | **ICD-9** | **ICD-10** |
| --- | --- | --- |
| Smoking related mortality |  |  |
| Cancer of buccal cavity, pharynx and oesophagus | 140-150 | C00-C15 |
| Cancer of larynx | 161 | C30-C32 |
| Cancer of trachea, bronchus, lung | 162-163;165 | C33-C34;C39 |
| Other chronic obstructive pulmonary diseases | 490-494;496 | J40-J44;J47 |
| Alcohol related mortality |  |  |
| Alcoholic cirrhosis of the liver and inflammation of the pancreas | 571.0-571.3, 577.0-577.1 | K70, K85-K86.0 |
| Accidental poisoning by alcohol | E860 | X45 |
| Alcoholic psychosis, dependence, abuse | 291,303,305.0 | F10 |

**Table S3** Relative risks comparing low educational level to high educational level for causes of amenable mortality by country

A. Infectious disease

| **Country** | **All infectious diseases**  **(ASMR)^*^** | | **All infectious diseases** | **TB** | **Pneumonia/influenza** | **Other infectious diseases** |
| --- | --- | --- | --- | --- | --- | --- |
|  | **Lower education** | **Higher education** | **RR (CI95)^**^** | **RR (CI95)** | **RR (CI95)** | **RR (CI95)** |
| **Western Europe** |  |  |  |  |  |  |
| Belgium | 31.38 | 23.16 | 1.15 (1.05-1.26) | 1.54 (1.17-2.04) | 1.69 (1.55-1.84) | 1.14 (1.04-1.27) |
| Switzerland | 32.96 | 23.35 | 1.74 (1.66-1.82) | 1.81 (1.39-3.34) | 1.71 (1.60-1.83) | 1.74 (1.62-1.86) |
| **Nordic countries** |  |  |  |  |  |  |
| Sweden | 22.94 | 15.63 | 1.46 (1.42-1.51) | 1.17 (1.02-1.33) | 1.52 (1.47-1.58) | 1.39 (1.31-1.47) |
| Finland | 48.23 | 30.99 | 1.59 (1.51-1.67) | 1.34 (1.12-1.60) | 1.71 (1.62-1.81) | 1.26 (1.12-1.42) |
| Denmark | 21.02 | 13.37 | 1.77 (1.63-1.93) | 2.49 (1.64-3.78) | 1.63 (1.45-1.82) | 1.88 (1.64-2.16) |
| Norway | 34.49 | 24.80 | 1.42 (1.35-1.49) | 1.41 (1.14-1.75) | 1.47 (1.39-1.56) | 1.27 (1.15-1.42) |
| **Southern Europe** |  |  |  |  |  |  |
| Italy (Turin) | 14.25 | 11.82 | 1.32 (1.08-1.61) | 2.42 (1.10-3.32) | 1.17 (0.92-1.49) | 1.50 (1.00-2.25) |
| Spain (Barcelona) | 27.82 | 25.71 | 1.33 (1.18-1.50) | 2.77 (1.59-3.25) | 1.20 (1.01-1.43) | 1.26 (1.05-1.51) |
| Spain (Madrid region) | 27.31 | 20.65 | 1.58 (1.26-1.98) | 1.92 (0.79-4.64) | 1.65 (1.16-2.35) | 1.49 (1.09-2.02) |
| Spain (Basque country) | 26.78 | 20.35 | 1.54 (1.32-1.80) | 2.19 (0.94-5.04) | 1.04 (0.75-1.43) | 1.74 (1.48-2.05) |
| **Baltic region** |  |  |  |  |  |  |
| Estonia | 83.79 | 31.01 | 2.19 (1.97-2.44) | 2.89 (2.36-3.55) | 2.06 (1.80-2.36) | 1.20 (0.78-1.84) |
| Lithuania | 91.74 | 20.79 | 3.53 (3.16-3.95) | 5.17 (4.41-6.06) | 2.80 (2.35-3.33) | 1.64 (1.21-2.21) |
| **Central and Eastern Europe** |  |  |  |  |  |  |
| Slovenia | 36.58 | 24.87 | 1.93 (1.77-2.10) | 3.47 (2.53-4.76) | 1.91 (1.72-2.11) | 1.42 (1.33-1.79) |
| Poland | 28.35 | 13.27 | 2.46 (2.34-2.58) | 6.01 (5.18-6.97) | 2.34 (2.20-2.48) | 1.62 (1.48-1.78) |
| Czech republic | 25.92 | 11.76 | 2.63 (2.44-2.85) | 5.02 (3.44-7.33) | 2.57 (2.36-2.79) | 2.28 (1.80-2.87) |
| Hungary | 18.49 | 7.27 | 2.94 (2.65-3.25) | 3.69 (3.07-4.42) | 3.14 (2.69-3.66) | 1.71 (1.39-2.10) |

*ASMR= age standardized mortality rate, ^**^ RR=relative risk (95% confidence interval)

B Cancer

| **Country** | **All amenable cancers (ASMR)^*^** | | | **Cervical cancer** | **Testicular cancer** | **Colorectal cancer** | **Hodgkin’s disease and leukaemia** |
| --- | --- | --- | --- | --- | --- | --- | --- |
|  | **Low** | **High** | **RR (CI95)^**^** | **RR (CI95)** | **RR (CI95)** | **RR (CI95)** | **RR** |
| **Western Europe** |  |  |  |  |  |  |  |
| Belgium | 51.88 | 49.95 | 1.10 (1.05-1.14) | 1.65 (1.36-2.01) | 0.94 (0.47-1.88) | 1.10 (1.05-1.16) | 1.03 (0.94-1.13) |
| Switzerland | 42.86 | 44.94 | 1.06 (1.02-1.10) | 2.04 (1.76-2.36) | 0.84 (0.51-1.39) | 1.04 (1.00-1.09) | 1.04 (0.96-1.13) |
| **Nordic countries** |  |  |  |  |  |  |  |
| Sweden | 43.07 | 39.69 | 1.09 (1.07-1.12) | 1.59 (1.45-1.74) | 1.82 (1.21-2.74) | 1.09 (1.07-1.12) | 1.01 (0.97-1.06) |
| Finland | 38.06 | 37.85 | 1.04 (1.00-1.09) | 1.69 (0.35-2.11) | 1.07 (0.51-2.22) | 1.01 (0.95-1.07) | 1.06 (0.96-1.16) |
| Denmark | 55.81 | 54.67 | 1.06 (1.00-1.10) | 1.88 (1.58-2.22) | 1.43 (0.86-2.37) | 1.01 (0.95-1.06) | 1.05 (0.94-1.18) |
| Norway | 60.65 | 56.72 | 1.08 (1.04-1.12) | 2.06 (1.80-2.35) | 0.57 (0.27-1.19) | 1.06 (1.02-1.11) | 0.92 (0.84-1.02) |
| **Southern Europe** |  |  |  |  |  |  |  |
| Italy (Turin) | 50.42 | 48.82 | 1.11 (1.00-1.23) | 3.06 (1.40-6.69) | 0.46 (0.14-1.52) | 1.13 (1.01-1.27) | 1.01 (0.82-1.24) |
| Spain (Barcelona) | 60.73 | 60.81 | 1.11 (1.03-1.20) | 1.68 (1.45-2.47) | 0.15 (0.13-1.77) | 1.13 (1.04-1.23) | 1.04 (0.88-1.22) |
| Spain (Madrid region) | 45.91 | 47.23 | 1.09 (0.94-1.27) | 2.70 (1.21-6.03) | 0.54 (0.03-9.68) | 1.02 (0.86-1.21) | 1.25 (0.90-1.75) |
| Spain (Basque country) | 35.24 | 43.73 | 0.97 (0.85-1.11) | 3.53 (1.49-8.36) | Not measurable | 0.96 (0.84-1.10) | 0.88 (0.67-1.15) |
| **Baltic region** |  |  |  |  |  |  |  |
| Estonia | 49.93 | 49.17 | 1.01 (0.91-1.11) | 1.51 (1.14-1.99) | 0.97 (0.54-3.99) | 0.97 (0.86-1.10) | 0.83 (0.66-1.04) |
| Lithuania | 63.06 | 48.22 | 1.19 (1.09-1.29) | 2.14 (1.76-2.62) | 2.20 (0.69-6.96) | 0.98 (0.88-1.09) | 1.16 (0.96-1.39) |
| **Central and Eastern Europe** |  |  |  |  |  |  |  |
| Slovenia | 58.93 | 65.68 | 1.01 (0.96-1.07) | 2.02 (1.65-2.48) | 0.67 (0.29-1.55) | 1.00 (0.94-1.07) | 0.97 (0.83-1.12) |
| Poland | 53.60 | 41.92 | 1.33 (1.29-1.37) | 1.70 (1.59-1.82) | 2.46 (1.67-3.61) | 1.24 (1.20-1.29) | 1.27 (1.20-1.35) |
| Czech republic | 75.35 | 54.18 | 1.48 (1.43-1.53) | 1.86 (1.64-2.11) | 1.81 (1.17-2.81) | 1.54 (1.47-1.60) | 1.24 (1.14-1.35) |
| Hungary | 75.66 | 61.49 | 1.33 (1.26-1.36) | 1.61 (1.43-1.80) | 1.50 (0.91-2.14) | 1.31 (1.25-1.36) | 1.26 (1.15-1.38) |

*ASMR=age standardized mortality rate, ^**^ RR=relative risk (95% confidence interval)

C. Cardiorespiratory conditions

| **Country** | **Cardio respiratory condtions**  **(ASMR)^*^** | | | **Ischemic heart disease** | **Cerebro vascular disease** | **Chronic rheumatic heart disease** | **Hypertension** | **Asthma** | **Other heart disease** |
| --- | --- | --- | --- | --- | --- | --- | --- | --- | --- |
|  | **Lower education** | **Higher education** | **RR (CI95)^**^** | **RR (CI95)** | **RR (CI95)** | **RR (CI95)** | **RR (CI95)** | **RR (CI95)** | **RR (CI95)** |
| **Western Europe** |  |  |  |  |  |  |  |  |  |
| Belgium | 330.39 | 256.53 | 1.39 (1.36-1.42) | 1.41 (1.37-1.45) | 1.32 (1.27-1.37) | 1.10 (0.73-1.65) | 1.44 (1.25-1.68) | 1.57 (0.36-1.82) | 1.43 (1.38-1.48) |
| Switzerland | 280.10 | 252.22 | 1.39 (1.37-1.41) | 1.31 (1.29-1.34) | 1.40 (1.35-1.45) | 1.46 (1.20-1.78) | 1.48 (1.39-1.58) | 1.55 (1.37-1.75) | 1.54 (1.49-1.59) |
| **Nordic countries** |  |  |  |  |  |  |  |  |  |
| Sweden | 335.74 | 244.29 | 1.43 (1.42-1.44) | 1.48 (1.47-1.50) | 1.31 (1.29-1.33) | 1.40 (1.25-1.55) | 1.57 (1.46-1.68) | 1.72 (1.58-1.87) | 1.37 (1.34-1.40) |
| Finland | 486.25 | 344.57 | 1.47 (1.45-1.49) | 1.52 (1.49-1.55) | 1.34 (1.30-1.39) | 1.23 (0.91-1.38) | 1.58 (1.39-1.79) | 1.76 (1.44-2.14) | 1.49 (1.42-1.57) |
| Denmark | 262.06 | 214.91 | 1.37 (1.34-1.41) | 1.44 (1.39-1.48) | 1.28 (1.22-1.34) | 1.03 (0.58-1.82) | 1.19 (1.05-1.35) | 1.88 (1.56-2.26) | 1.35 (1.28-1.42) |
| Norway | 416.00 | 306.80 | 1.44 (1.42-1.46) | 1.50 (1.47-1.53) | 1.32 (1.28-1.36) | 1.39 (1.16-1.66) | 1.49 (1.36-1.63) | 2.04 (1.85-2.26) | 1.39 (1.33-1.44) |
| **Southern Europe** |  |  |  |  |  |  |  |  |  |
| Italy (Turin) | 257.37 | 230.71 | 1.25 (1.20-1.32) | 1.26 (1.17-1.36) | 1.26 (1.16-1.38) | 1.13 (0.77-1.66) | 1.10 (0.93-1.29) | 1.05 (0.64-1.72) | 1.31 (1.19-1.45) |
| Spain (Barcelona) | 285.13 | 272.88 | 1.21 (1.17-1.26) | 1.17 (1.11-1.24) | 1.28 (1.19-1.38) | 1.40 (1.08-1.80) | 1.18 (0.96-1.44) | 1.90 (1.24-2.91) | 1.20 (1.11-1.30) |
| Spain (Madrid region) | 175.58 | 177.01 | 1.15 (1.07-1.24) | 1.02 (0.91-1.46) | 1.11 (0.93-1.32) | 2.26 (1.24-4.13) | 0.86 (0.53-1.38) | 2.49 (0.75-8.28) | 1.37 (1.71-1.60) |
| Spain (Basque country) | 149.59 | 165.30 | 1.03 (0.97-1.10) | 1.01 (0.93-1.11) | 0.97 (0.87-1.09) | 1.13 (0.72-1.77) | 1.27 (0.89-1.81) | 0.90 (0.44-1.86) | 1.15 (1.01-1.31) |
| **Baltic region** |  |  |  |  |  |  |  |  |  |
| Estonia | 786.51 | 498.34 | 1.54 (1.49-1.58) | 1.52 (0.47-1.58) | 1.53 (1.45-1.62) | 1.34 (1.00-1.81) | 1.60 (1.40-1.83) | 1.71 (1.24-2.36) | 1.78 (1.55-2.04) |
| Lithuania | 677.26 | 374.36 | 1.66 (1.61-1.71) | 1.63 (1.57-1.70) | 1.64 (1.55-1.74) | 1.84 (1.49-2.28) | 1.06 (0.88-1.27) | 2.04 (1.34-3.02) | 2.02 (1.85-2.09) |
| **Central and Eastern Europe** | |  |  |  |  |  |  |  |  |
| Slovenia | 440.45 | 375.40 | 1.44 (1.41-1.47) | 1.16 (1.12-1.20) | 1.74 (1.67-1.82) | 1.35 (1.11-1.63) | 1.43 (1.29-1.58) | 1.65 (1.29-2.10) | 1.72 (1.63-1.81) |
| Poland | 459.64 | 242.49 | 2.02 (1.99-2.04) | 1.86 (1.83-1.89) | 2.06 (2.01-2.08 | 1.93 (1.75-2.13) | 2.27 (2.13-2.41) | 2.51 (2.21-2.87) | 2.23 (2.18-2.80) |
| Czech republic | 442.65 | 242.62 | 2.16 (2.12-2.20) | 2.19 (2.14-2.45) | 2.24 (2.16-2.32) | 1.77 (1.48-2.11) | 2.21 (2.00-1.45) | 2.39 (1.85-3.09) | 1.90 (1.82-1.99) |
| Hungary | 591.16 | 305.30 | 2.22 (2.18-2.25) | 2.04 (1.99-2.09) | 2.40 (2.32-2.47) | 2.08 (1.67-2.44) | 2.44 (2.27-2.62) | 2.51 (1.94-3.24) | 2.54 (2.42-2.68) |

*ASMR=age standardized mortality rate, ^**^ RR=Relative Risk (95% confidence interval)

D. Gastrointestinal conditions

| **Country** | **Total gastrointestinal conditions**  **(ASMR)^**^** | | | **Appendicitis, hernia and peptic ulcer** | **Cholecystitis and -lithiasis** |
| --- | --- | --- | --- | --- | --- |
|  | **Lower education** | **Higher education** | **RR (CI95)^*^** | **RR (CI95)** | **RR (CI95)** |
| **Western Europe** |  |  |  |  |  |
| Belgium | 7.87 | 5.57 | 1.47 (1.30-1.67) | 1.48 (1.28-1.72) | 1.44 (1.10-1.89) |
| Switzerland | 6.82 | 4.94 | 1.53 (1.39-1.69) | 1.51 (1.35-1.69) | 1.62 (1.30-2.02) |
| **Nordic countries** |  |  |  |  |  |
| Sweden | 8.79 | 5.41 | 1.64 (1.56-1.73) | 1.67 (0.58-1.77) | 1.52 (1.34-1.71) |
| Finland | 14.89 | 9.54 | 1.64 (1.50-1.80) | 1.65 (1.49-1.82) | 1.66 (1.33-2.06) |
| Denmark | 13.94 | 9.63 |  |  |  |
| Norway | 11.02 | 7.48 | 1.52 (1.39-1.66) | 1.56 (1.41-1.72) | 1.39 (1.14-1.70) |
| **Southern Europe** |  |  |  |  |  |
| Italy (Turin) | 8.47 | 5.60 | 1.77 (1.32-2.38) | 1.67 (1.21-2.30) | 2.38 (1.14-4.95) |
| Spain (Barcelona) | 8.32 | 7.42 | 1.26 (1.01-1.57) | 1.18 (0.90-1.53) | 1.00 (0.41-2.45) |
| Spain (Madrid region) | 4.97 | 5.93 | 1.17 (0.70-1.96) | 1.26 (0.67-2.36) | 1.40 (0.83-2.37) |
| Spain (Basque country) | 3.95 | 3.33 | 1.29 (0.85-1.96) | 1.11 (0.69-1.81) | 1.88 (0.81-4.35) |
| **Baltic region** |  |  |  |  |  |
| Estonia | 16.85 | 9.07 | 1.69 (1.37-2.10) | 1.75 (1.39-2.21) | 1.40 (0.83-2.37) |
| Lithuania | 14.47 | 8.41 | 1.60 (1.31-1.95) | 1.62 (1.30-2.03) | 1.49 (0.96-2.33) |
| **Central and Eastern Europe** |  |  |  |  |  |
| Slovenia | 16.22 | 11.64 | 1.63 (1.44-1.86) | 1.68 (1.46-1.93) | 1.42 (1.05-1.93) |
| Poland | 11.11 | 5.46 | 2.31 (2.14-2.49) | 2.28 (2.10-2.48) | 2.46 (1.97-3.06) |
| Czech republic | 11.28 | 5.26 | 2.43 (2.17-2.73) | 2.48 (2.19-2.81) | 2.11 (1.57-2.83) |
| Hungary | 17.25 | 9.39 | 2.12 (1.93-2.34) | 2.21 (0.97-2.25) | 1.65 (1.31-2.09) |

*age standardized mortality rate, ^**^Relative Risk (95% confidence interval)

**Table S4a.** Correlation between inequalities in causes of amenable mortality and inequalities in use of health care

|  | **Use of health care** | | | | | | | |
| --- | --- | --- | --- | --- | --- | --- | --- | --- |
| **Cause of death** | **GP visit** | **Visit Specialist** | **Visit any doctor** | **Hospitalization** | **Use of medication** | **Use of prescribed medication** | **Cholesterol screening** | **BP screening** |
| Number of observations | 7 | 6 | 11 | 8 | 8 | 5 | 8 | 8 |
| All cause mortality | −0.668 | −0.767 | -0.773* | −0.159 | −0.585 | 0.869 | −0.151 | 0.151 |
| Non amenable causes of death | −0.642 | −0.776 | -0.760* | −0.220 | −0.686 | 0.958* | −0.026 | 0.197 |
| **Infectious disease** |  |  |  |  |  |  |  |  |
| Infectious disease | −0.835* | −0.741 | -0.767* | −0.044 | −0.532 | 0.860 | 0.284 | −0.097 |
| Tuberculosis | −0.804 | −0.652 | −0.584* | −0.417 | −0.693 | 0.946* | 0.489 | 0.315 |
| pneumonia/influenza | −0.637 | 0.729 | -0.783* | −0.065 | −0.566 | 0.803 | −0.088 | −0.131 |
| Other infectious disease | −0.603 | −0.763 | −0.439 | −0.150 | −0.608 | 0.736 | 0.245 | −0.762* |
| **Cancer** |  |  |  |  |  |  |  |  |
| Amenable cancer | −0.465 | −0.862* | −0.538 | −0.359 | −0.775* | 0.989* | −0.282 | 0.131 |
| Cervical cancer | 0.302 | 0.117 | −0.176 | −0.248 | −0.048 | −0.190 | 0.349 | 0.031 |
| Testicular cancer | −0.640 | −0.815* | −0.643* | −0.053 | −0.618 | 0.786 | 0.162 | 0.345 |
| Colorectal cancer | −0.409 | −0.819* | -0.384 | −0.378 | −0.735* | 0.955* | −0.540 | 0.051 |
| Hodgkin's disease and leukaemia | −0.207 | −0.819* | -0.396 | −0.571 | −0.908* | 0.910* | −0.203 | 0.316 |
| **Cardiorespiratory conditions** | −0.642 | −0.799 | −0.749* | −0.102 | −0.606 | 0.900* | −0.296 | 0.209 |
| Ischaemic heart disease | −0.670 | −0.824* | −0.622* | −0.032 | −0.554 | 0.872 | −0.254 | 0.358 |
| Cerebrovascular disease | −0.632 | −0.786 | −0.752* | −0.216 | −0.674 | 0.952* | −0.297 | 0.192 |
| Chronic rheumatic heart disease | −0.685 | −0.740 | −0.677* | −0.225 | −0.450 | 0.787 | −0.039 | −0.047 |
| Hypertension | −0.527 | −0.686 | −0.507 | −0.107 | −0.543 | 0.827 | −0.649 | 0.164 |
| Asthma | −0.632 | −0.770 | −0.426 | −0.207 | −0.296 | 0.442 | −0.223 | 0.149 |
| Heartfailure | −0.671 | −0.720 | −0.216 | −0.005 | −0.518 | 0.921* | −0.203 | −0.238 |
| **Gastrointestinal conditions** |  |  |  |  |  |  |  |  |
| Total amenable gastrointestinal conditions | −0.487 | −0.639 | −0.668* | −0.036 | −0.667 | 0.998* | −0.254 | 0.548 |
| Appendicitis, hernia, peptic ulcer | −0.518 | -.630 | −0.690 | −0.011 | −0.608 | 0.989* | −0.300 | 0.455 |
| Cholecystitis and lithiasis | 0.097 | −0.053 | −0.078 | 0.222 | −0.454 | 0.500 | 0.133 | 0.681 |

*significant correlation, p<0.05

**Table S4b**. Correlation between inequalities in causes of amenable mortality and inequalities in behavioural risk factors

|  | **Behavioural risk factors** | | **Causes of death related to behavioural risk factors** | |
| --- | --- | --- | --- | --- |
|  | **Smoking** | **Overweight** | **Smoking related deaths** | **Alcohol related deaths** |
| **Nr of observations** | 12 | 11 | 14 | 14 |
| **Cause of death** |  |  |  |  |
| All cause mortality | 0.895* | −0.093 | 0.839* | 0.534* |
| Non amenable causes of death | 0.846* | 0.040 | 0.811* | 0.634* |
| **Infectious disease** |  |  |  |  |
| Infectious disease | 0.639* | −0.206 | 0.615* | 0.663* |
| TB | 0.552 | 0.053 | 0.547* | 0.788* |
| pneumonia/influenza | 0.816* | −0.115 | −0.734* | 0.486 |
| Other infectious disease | 0.644* | 0.206 | 0.363 | 0.478 |
| **Cancer** |  |  |  |  |
| Amenable cancer | 0.748* | 0.143 | 0.473 | 0.563* |
| Cervical cancer | −0.281 | 0.482 | −0.459 | 0.136 |
| Testicular cancer | 0.686* | −0.159 | 0.629* | 0.547 |
| Colorectal cancer | 0.598* | 0.231 | 0.297 | 0.359 |
| Hodgkin's disease and leukaemia | 0.661* | 0.266 | 0.290 | 0.385 |
| **Cardiorespiratory conditions** | 0.906* | −0.026 | 0.815* | 0.409 |
| Ischaemic heart disease | 0.851* | −0.026 | 0.897* | 0.367 |
| Cerebrovascular disease | 0.867* | 0.036 | 0.732* | 0.554* |
| Chronic rheumatic heart disease | 0.646* | −0.322 | 0.286 | 0.306 |
| Hypertension | 0.782* | 0.022 | 0.675* | 0.281 |
| Asthma | 0.840* | −0.325 | 0.512 | 0,267 |
| Heartfailure | 0.064 | −0.103 | −0.137 | 0.326 |
| **Gastrointestinal conditions** |  |  |  |  |
| Total amenable gastrointestinal conditions | 0.723* | 0.256 | 0.789* | 0.473 |
| Appendicitis, hernia, peptic ulcer | 0.763* | 0.145 | 0.798* | 0.411 |
| Cholecystitis and -lithiasis | 0.144 | 0.678* | 0.194 | 0.277 |

*significant correlation, p<0.05
